# Pulmonary vasoreactivity in spontaneously hypertensive rats - Effects of endothelin-1 and leptin

**DOI:** 10.1186/1465-9921-15-12

**Published:** 2014-02-05

**Authors:** Samantha Gomart, Cécile Damoiseaux, Pascale Jespers, Martine Makanga, Nathalie Labranche, Stéphanie Pochet, Charles Michaux, Guy Berkenboom, Robert Naeije, Kathleen McEntee, Laurence Dewachter

**Affiliations:** 1 Laboratory of Physiology, Faculty of Medicine, Université Libre de Bruxelles, Lennik road 808, 1070 Brussels, Belgium; 2 Laboratory of Physiology and Pharmacology, Faculty of Pharmacy, Université Libre de Bruxelles, Brussels, Belgium; 3 Faculty of Medicine, Université Libre de Bruxelles, Brussels, Belgium; 4 Department of Cardiology, Erasme Academic Hospital, Université Libre de Bruxelles, Brussels, Belgium

**Keywords:** Systemic hypertension, Pulmonary circulation, Vascular reactivity, Leptin, Endothelin-1

## Abstract

**Background:**

Systemic hypertension may be associated with an increased pulmonary vascular resistance, which we hypothesized could be, at least in part, mediated by increased leptin.

**Methods:**

Vascular reactivity to phenylephrine (1 μmol/L), endothelin-1 (10 nmol/L) and leptin (0.001–100 nmol/L) was evaluated in endothelium-intact and -denuded isolated thoracic aorta and pulmonary arteries from spontaneously hypertensive *versus* control Wistar rats. Arteries were sampled for pathobiological evaluation and lung tissue for morphometric evaluation.

**Results:**

In control rats, endothelin-1 induced a higher level of contraction in the pulmonary artery than in the aorta. After phenylephrine or endothelin-1 precontraction, leptin relaxed intact pulmonary artery and aortic rings, while no response was observed in denuded arteries. Spontaneously hypertensive rats presented with increased reactivity to phenylephrine and endothelin-1 in endothelium-intact pulmonary arteries. After endothelin-1 precontraction, endothelium-dependent relaxation to leptin was impaired in pulmonary arteries from hypertensive rats. In both strains of rats, aortic segments were more responsive to leptin than pulmonary artery. In hypertensive rats, pulmonary arteries exhibited increased pulmonary artery medial thickness, associated with increased expressions of preproendothelin-1, endothelin-1 receptors type A and B, inducible nitric oxide synthase and decreased endothelial nitric oxide synthase, together with decreased leptin receptor and increased suppressor of cytokine signaling 3 expressions.

**Conclusions:**

Altered pulmonary vascular reactivity in hypertension may be related to a loss of endothelial buffering of vasoconstriction and decreased leptin-induced vasodilation in conditions of increased endothelin-1.

## Background

It has long been known that pulmonary vascular resistance (PVR) may be increased in patients with systemic hypertension, even when left ventricular filling pressures are within the limits of normal [1]. Guazzi et al. hypothesized that this would be related to shared abnormal smooth muscle calcium handling mechanisms in the systemic and pulmonary resistive vessels [2]. In support of this notion, these authors reported an enhanced pulmonary vascular reactivity to hypoxia in patients with uncomplicated systemic hypertension [3]. However, additional factors could also be implicated such as decreased expression of nitric oxide (NO) and increased expression of endothelin-1 [4,5] related to latent left heart failure at rest, but increased left ventricular diastolic pressures at exercise, as a cause of intermittent upstream transmission of mechanical stress with endothelial damage to the pulmonary circulation. Another factor involved could be leptin, a peptide hormone secreted by adipocytes, which has been shown to be increased in hypertensive patients even after adjustment for major confounders, including obesity [6,7]. Leptin is currently thought to contribute to systemic hypertension by a combination of mechanisms including sympathetic nervous system activation [8,9], overproduction of endothelin-1 [10,11] and decreased release of NO [12]. In patients with pulmonary arterial hypertension, there is an increase in circulating leptin which may contribute to inflammatory changes in the pulmonary circulation and thereby increase PVR [13].

We, therefore, explored and compared the vascular reactivity to endothelin-1 and leptin in isolated systemic and pulmonary arteries from spontaneously hypertensive (SHR) compared to control Wistar rats. The results are compatible with the notion of abnormal pulmonary endothelial function involving leptin and endothelin-1 control in systemic hypertension.

## Methods

The present study was approved by the Institutional Animal Care and Use Committee of the Faculty of Medicine of the *Université Libre de Bruxelles* (Brussels, Belgium) and was conducted in accordance with the “Guide for the Care and Use of Laboratory Animals” published by the US National Institutes of Health (NIH publication no. 85 – 23, revised 1996).

### Animals and sample preparation

Experiments were conducted in 18-week-old male spontaneously hypertensive (SHR) and control Wistar rats (Janvier, Le Genest Saint-Isle, France), weighing respectively 340 ± 4 and 441 ± 10 g. During a one-week acclimatization period, the rats were housed in a temperature (21°C)- and relative humidity (60%)-controlled room and exposed to a 12-hour light and dark cycle. Standard rat chow and tap water were given *ad libitum*.

After euthanasia of the animals with carbon dioxide, thoracic aorta and pulmonary artery (taken after the first branch point of the common pulmonary trunk) were carefully excised and cleaned of blood. Adhesive fat and connective tissue were removed. Sampled artery sections were immediately snap-frozen and stored at −80°C for biological evaluation, or placed in Krebs-Henseleit solution (118.1 mmol/L NaCl; 4.7 mmol/L KCl; 1.2 mmol/L MgSO_4_; 1.2 mmol/L KH_2_PO_4_; 2.5 mmol/L CaCl_2_; 25 mmol/L NaHCO_3_; 5.1 mmol/L glucose) for vasoreactivity experiments.

Thoracic aortic and pulmonary artery segments were harvested, snap-frozen in liquid nitrogen and stored at −80°C for biological experiments. Pulmonary tissue samples were immediately harvested and embedded in paraffin for morphometric evaluation.

### Vascular reactivity

Under a dissecting microscope, thoracic aorta and pulmonary artery were cut into segments of ~3 mm-length and 2.3 ± 0.1 and 1.8 ± 0.2 mm internal diameter respectively. During dissection, care was taken to protect the endothelial lining in endothelium-intact rings. In half of the rings, the endothelium was removed by rubbing the inner intimal surface of the vascular lumen with a surgical steel rod to obtain endothelium-denuded rings.

Thoracic aortic and pulmonary artery rings were mounted on stainless steel hooks in 5 mL-organ baths filled with Krebs-Henseleit solution bubbled with 95% O_2_ and 5% CO_2_ and maintained at 37°C. One of the steel hooks was anchored in the chamber; the other was connected to a force transducer for continuous recording of isometric tension by a personal computer and a chart recorder (EMKA Technologies, Paris, France). The rings were placed under a resting tension of 1000 mg and 600 mg for thoracic aortic and pulmonary artery rings respectively and were allowed to equilibrate for 60 minutes. Krebs solution was changed every 20 minutes.

Thoracic aortic and pulmonary artery rings were contracted with 80 mmol/L KCl (Sigma-Aldrich, Bornem, Belgium) to assess their contractility. Subsequently, segments were contracted with phenylephrine hydrochloride (1 μmol/L; Sigma-Aldrich, Bornem, Belgium) and acetylcholine chloride (0.001 to 10 μmol/L; Sigma-Aldrich) was added to functionally confirm the presence of the endothelium. If the tension of the vessel after administration of acetylcholine (10 μmol/L) was under 20% or above 80% of the phenylephrine induced precontraction, the vessels were considered respectively as endothelium-intact or -denuded. The vessels that did not belong to any of these two groups were excluded from the present study.

After washout period allowing return to basal vascular tone, rat leptin (Sigma-Aldrich) was tested between 0.001 to 100 nmol/L (incubated during 2 minutes 30 seconds each) in thoracic aortic and pulmonary artery rings preconstricted with phenylephrine (1 μmol/L). The washout period was repeated to allow the vessel rings to return to their basal vascular tone. Subsequently, rat leptin (100 nmol/L) was tested in rings preconstricted with endothelin-1 (10 nmol/L; Sigma-Aldrich), with tension recording every 2.5 minutes during a total of 25 minutes.

The phenylephrine (1 μmol/L) and endothelin-1 (10 nmol/L) concentrations were chosen from complete concentration-response curves (from 10^-9^ to 10^-5^ mol/L in log increments; data not shown). Selected concentrations induced strong artery segment constriction (> 100 mg for the pulmonary artery and > 1000 mg for the thoracic aorta), but were inferior to the concentrations inducing maximal constriction.

In order to evaluate the role played by the endothelium in the observed variations of vascular tone, experiments were carried out in endothelium-intact and -denuded aortic and pulmonary artery segments.

### Morphometry—Immunohistochemistry

Pulmonary arterial morphometry was performed as previously described [14,15]. Medial thickness (MT) was related to arterial size with the following formula:%MT = (2MT/ED) × 100 and performed by counting at least 50 pulmonary arteries per lung section from each rat.

### Real-time quantitative polymerase chain reaction (RTQ-PCR)

Total RNA was extracted from snap-frozen thoracic aortic and pulmonary artery segments by homogenization according to the method of Chomczynski and Sacchi [16], using TRIzol reagent (Invitrogen, Merelbeke, Belgium) and further purified using RNeasy® Mini kit (QIAGEN S.A., Hilden, Germany) according to the manufacturer’s instructions. RNA concentration was determined by spectrophotometry (Nanodrop® ND-1000, Isogen life science, De Meern, The Netherlands) and RNA integrity assessed by visual inspection of GelRed (Biotium, Hayward, California)-stained agarose gels.

Reverse transcription was carried out using SuperScript^TM^ II Reverse Transcriptase (Invitrogen), according to the manufacturer’s instructions.

For RTQ-PCR, sense and anti-sense primers were designed using Primer3 program for *rattus norvegicus* leptin, leptin receptor, suppressor of cytokine signaing 3 (SOCS3), preproendothelin-1 (PPET-1), endothelin-converting enzyme 1 (ECE-1), endothelin receptor type A (ET_A_), endothelin receptor type B (ET_B_), endothelial nitric oxide synthase (eNOS), inducible nitric oxide synthase (iNOS) and hypoxanthine-guanine phosphoribosyltransferase (HPRT1) mRNA sequences amplification (Table 1). To avoid inappropriate amplification of residual genomic DNA, intron-spanning primers were selected when exon sequences were known and a BLAST analysis was run to check if primer pairs were only matching at the sequence of interest. For each sample, amplification reaction was performed in triplicate using SYBRGreen PCR Master Mix (Quanta Biosciences, Gaithersburg, Maryland), specific primers and diluted template cDNA. Result analysis was performed using an iCycler system (BioRad Laboratories, Nazareth Eke, Belgium). Relative quantification was achieved with the comparative 2^-∆∆Ct^ method by normalization with the housekeeping gene, HPRT1 [17].

**Table 1:** **Primers used for real-time quantitative polymerase chain reaction in *rattus norvegicus *** **thoracic aorta and pulmonary arteries**

**Genes**		**Sequences**
Hypoxanthine phosphoribosyltransferase 1 (HPRT1)	Sense	5′ – ACAGGCCAGACTTTGTTGGA – 3′
	Antisense	5′ – TCCACTTTCGCTGATGACAC – 3′
Endothelin receptor type A (ETA)	Sense	5′ – CTGGTGGCTCTTTGGATTCT – 3′
	Antisense	5′ – GCTCCCATTCCTTCTGTTGA – 3′
Endothelin receptor type B (ETB)	Sense	5′ – CGATTGTATCATGCCTCGTG – 3′
	Antisense	5′ – GGGACCATTTCTCATGCACT – 3′
Leptin	Sense	5′ – CCTGTGGCTTTGGTCCTATC – 3′
	Antisense	5′ – ATACCGACTGCGTGTGTGAA – 3′
Leptin receptor	Sense	5′ – GGACAGCCAAACAAAAGCAC – 3′
	Antisense	5′ – TATCCGAGACGATTTCAGCA – 3′
Endothelial nitric oxide synthase (eNOS)	Sense	5′ – GGTATTTGATGCTCGGGACT – 3′
	Antisense	5′ – TGATGGCTGAACGAAGATTG – 3′
Inducible nitric oxide synthase (iNOS)	Sense	5′ – GTTTCCCCCAGTTCCTCACT – 3′
	Antisense	5′ – CTCTCCATTGCCCCAGTTT –5′
Preproendothelin-1 (PPET-1)	Sense	5′ – CAGACCAAGGGAACAGATGC – 3′
	Antisense	5′ – ACGCCTTTCTGCATGGTACT – 3′
Endothelin-converting enzyme 1 (ECE1)	Sense	5′ – GCCCACCAAGAACGAGATT – 3′
	Antisense	5′ – GACCCCGATACCAAAGT – 3′
Suppressor of cytokine signaling 3 (SOCS3)	Sense	5′ – TGCAGGAGAGCGGATTCTAC – 3′
	Antisense	5′ – AGCTGTCGCCCATAAGAAAG – 3′

### Statistical analysis

All values are expressed as mean ± standard error of the mean (SEM). Relaxations to acetylcholine or leptin were expressed as the percentage of the maximal contractile response developed by phenylephrine or endothelin-1. The repeated relaxation response measurements in control Wistar and spontaneously hypertensive rats were analyzed with a mixed linear model to assess the effect of leptin concentrations (from 0.001 to 100 nmol/L by step of 0.1 mol/L) or the effect of time of a leptin concentration of 100 nmol/L (from 5 to 25 minutes by step of 2.5 minutes) and to compare thoracic aorta and pulmonary artery, to compare endothelium-intact and -denuded arteries and to test the interaction between these three factors. The repeated relaxation response measurements in thoracic aorta and pulmonary artery were also analyzed with a mixed linear model to assess the effect of leptin concentrations or the effect of time of a leptin concentration of 100 nmol/L and to compare spontaneously hypertensive and control rat lines, to compare endothelium-intact and -denuded arteries and to test the interaction between these three factors. Differences in maximal contraction to phenylephrine or endothelin-1, RT-QPCR and morphometric data were tested by Student *t* tests. A value of p < 0.05 was considered statistically significant; n represents the number of individual data.

## Results

### Vascular reactivity: responses to phenylephrine and endothelin-1

**Figure 1:**
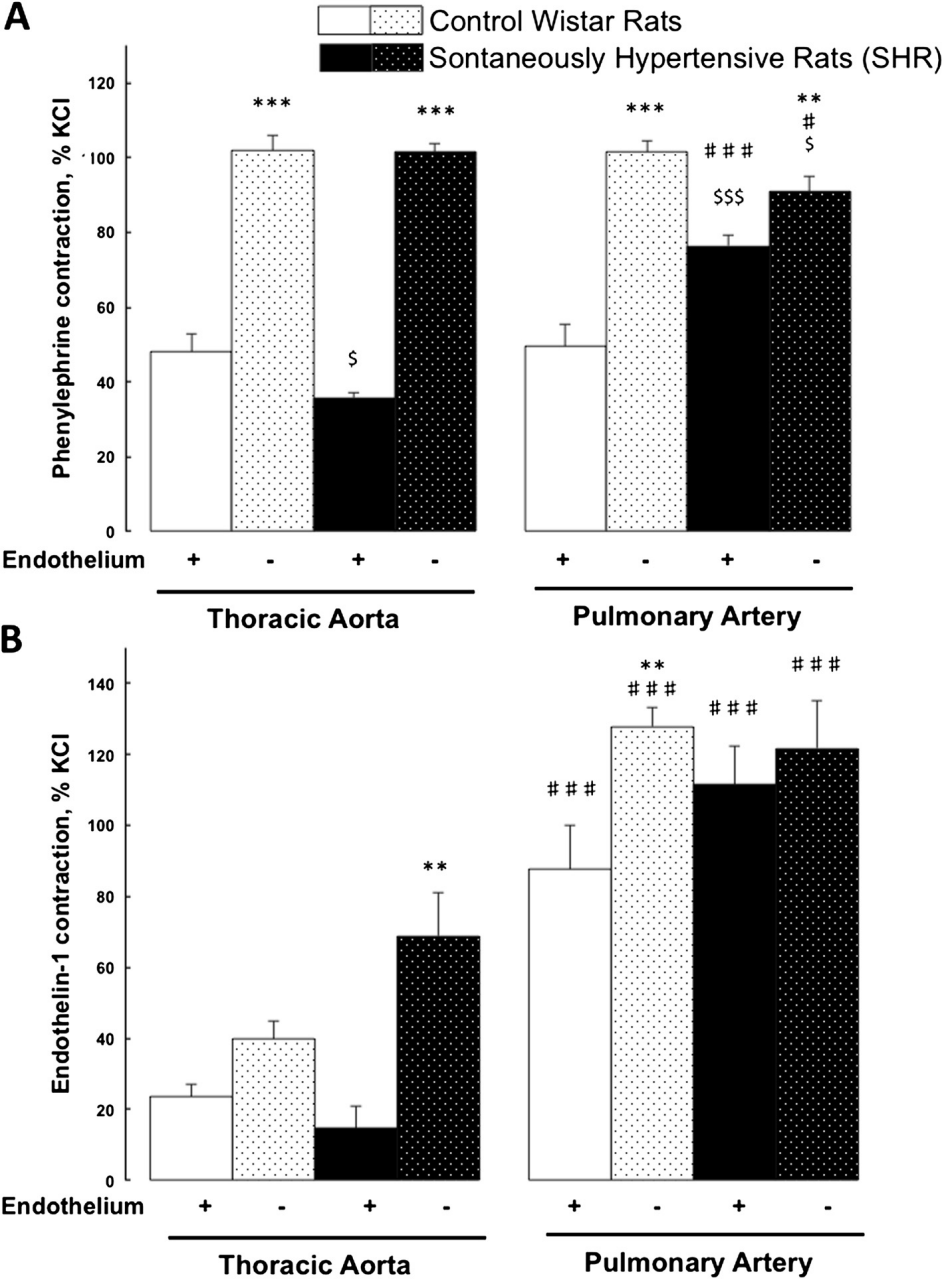
**Maximal contractile responses to phenylephrine and endothelin-1 in control Wistar and spontaneously hypertensive rats.** Comparison of contractile responses of endothelium-denuded (−) and –intact (+) thoracic aortic and pulmonary artery rings to phenylephrine (1 μmol/L) **(A)** and endothelin-1 (10 nmol/L) **(B)**. Thoracic aortic and pulmonary artery segments are collected in control Wistar rats (white bars; n = 7-13) and in spontaneously hypertensive rats (black bars; n = 10-15). Contractile responses are expressed as the percentage of the maximal contractile response to 80 mmol/L KCl. Results are expressed as means ± SEM. ** 0.001 < p < 0.01, *** p < 0.001 compared to the corresponding endothelium-intact (+) artery; $ 0.01 < p < 0.05, $$$ p < 0.001 compared to the corresponding artery from the control group; ♯ 0.01 < p < 0.05, ♯♯♯ p < 0.001 compared to the thoracic artery in the same strain of rats.

In control rats, the maximal contractile responses to phenylephrine (1 μmol/L) were comparable in pulmonary artery and thoracic aortic rings and were enhanced in endothelium-denuded rings (Figure 1A). After phenylephrine precontraction, acetylcholine (dose range tested from 0.001 to 10 μmol/L) induced a similar concentration-dependent relaxation in endothelium-intact pulmonary artery and thoracic aortic rings. This relaxation was similarly abolished in endothelium-denuded pulmonary artery and aortic rings. The maximal relaxation responses to acetylcholine (10 μmol/L) in endothelium-intact pulmonary artery and aorta were, respectively, 95 ± 3% and 86 ± 2% (not significant, p > 0.05) and in endothelium-denuded pulmonary artery and aorta 2 ± 1% and 1 ± 1%, (not significant, p > 0.05). In endothelium-intact and -denuded artery rings, endothelin-1 (10 nmol/L) induced a higher level of contraction in pulmonary artery compared to aorta (Figure 1B). In endothelium-denuded pulmonary artery, the contractile response to endothelin-1 (10 nmol/L) was increased compared to the one observed in endothelium-intact pulmonary artery (Figure 1B).

In spontaneously hypertensive rats (SHR), the maximal contractile response to phenylephrine (1 μmol/L) was higher in pulmonary artery compared to thoracic aortic rings. The enhanced contractile response observed in endothelium-denuded compared to –intact artery segments was abolished in SHR (Figure 1A). The maximal pulmonary artery and thoracic aortic relaxation responses to acetylcholine (10 μmol/L) after phenylephrine (1 μmol/L) precontraction were of similar magnitude in SHR compared to controls. This was observed in endothelium-intact (92 ± 2% in SHR versus 95 ± 3% in controls; not significant, p > 0.05) and endothelium-denuded (1 ± 1% in SHR versus 2 ± 1% in controls; not significant, p > 0.05) pulmonary artery rings.

As illustrated in Figure 1, the maximal contractile responses to phenylephrine (1 μmol/L) and to endothelin-1 (10 nmol/L) were respectively significantly and non significantly (but with a strong tendency; p = 0.058) enhanced in endothelium-intact pulmonary artery rings from SHR compared to controls. These maximal contractions to phenylephrine and to endothelin-1 were respectively 51% and 32% higher in endothelium-denuded compared to -intact pulmonary artery rings from controls, while these differences were respectively reduced to 16% or even abolished in SHR (Figure 1). These data suggest that the endothelial negative modulation of vasoconstriction was decreased or even abolished in SHR.

### Vasoreactivity of thoracic aorta and pulmonary artery: responses to leptin

**Figure 2:**
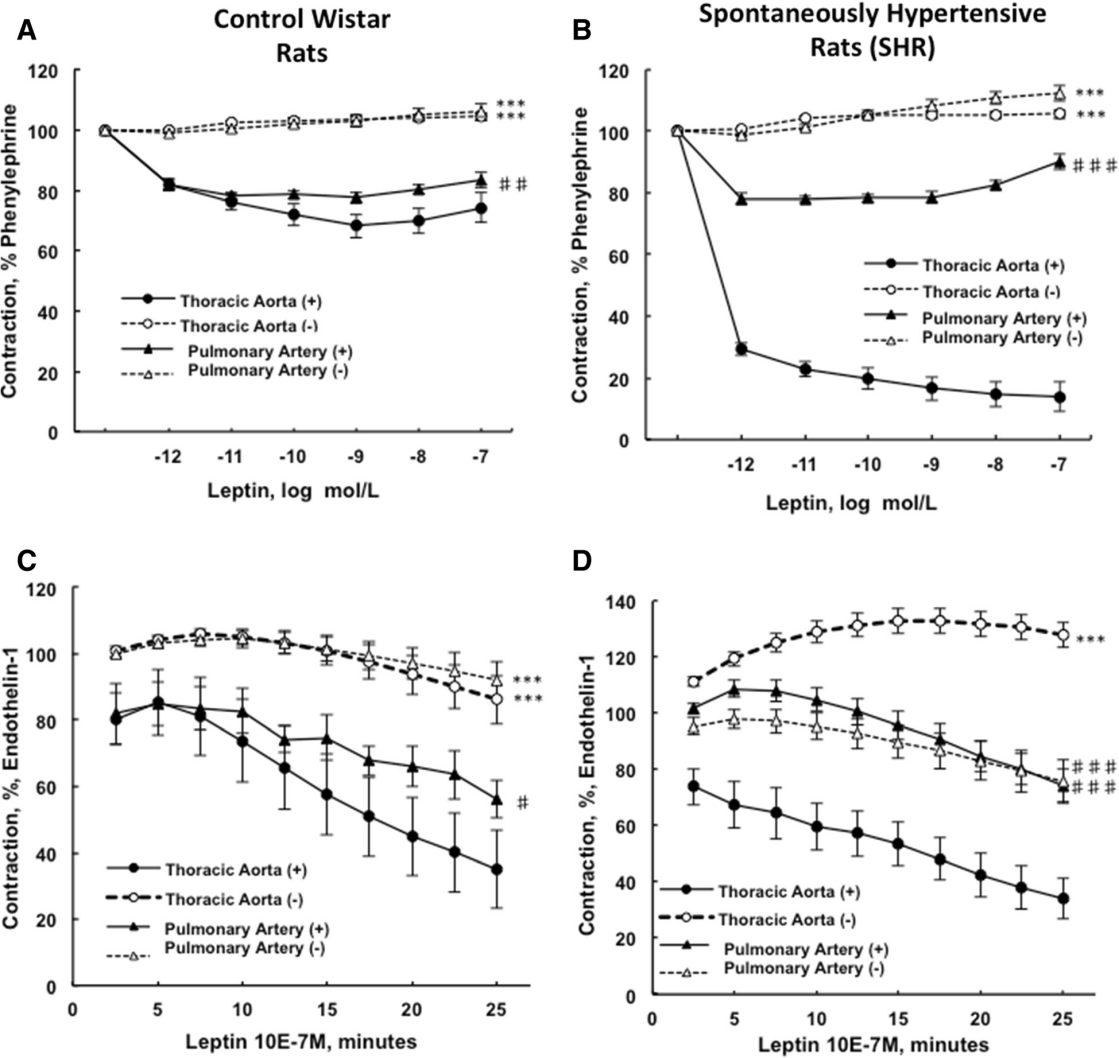
**Concentration-response curves to leptin after phenylephrine precontraction in pulmonary artery compared to thoracic aortic segments.** The endothelium-denuded (−) and –intact (+) thoracic aortic and pulmonary artery rings collected from control Wistar **(A)** and spontaneously hypertensive rats **(B)** were precontracted with phenylephrine (1 μmol/L) before cumulative addition of leptin (0.001 to 100 nmol/L). Relaxation responses are expressed as the percentages of the maximal tension response obtained with 1 μmol/L phenylephrine. Response curves to leptin after endothelin-1 precontraction in pulmonary artery compared to thoracic aortic segments. The endothelium-denuded (−) and –intact (+) thoracic aortic and pulmonary artery rings from control Wistar rats **(C)** and spontaneously hypertensive rats **(D)** were precontracted with endothelin-1 (10 nmol/L) before the addition of leptin (100 nmol/L). Relaxation responses are expressed as the percentages of the maximal tension response obtained with 10 nmol/L endothelin-1. Thoracic aortic and pulmonary artery segments are collected in control Wistar rats (n = 7-13) and in spontaneously hypertensive rats (n = 10-15). Results are expressed as means ± SEM. *** p < 0.001 compared to the corresponding endothelium-intact (+) artery; ♯ 0.01 < p < 0.05, ♯♯ 0.001 < p < 0.01, ♯♯♯ p < 0.001 compared to the thoracic artery in the same strain of rats.

As illustrated in Figure 2A and B, leptin (tested from 0.001 to 100 nmol/L) induced a concentration-dependent relaxation after phenylephrine (1 μmol/L) precontraction, rapidly reaching a plateau in endothelium-intact pulmonary artery and aortic rings from control and hypertensive rats. This vasodilating effect was more pronounced in aorta than in pulmonary arteries in both strains of rats. No response to leptin was observed in endothelium-denuded arteries.

In controls, leptin (100 nmol/L) induced the relaxation, of endothelium-intact pulmonary artery and aortic rings after endothelin-1 (10 nmol/L) precontraction, while this was not observed in denuded rings (Figure 2C). In SHR, leptin (100 nmol/L) also induced the relaxation of endothelium-intact pulmonary artery and aortic rings after endothelin-1 (10 nmol/L) precontraction, and this effect was abolished in denuded aortic rings only. Indeed, endothelium-intact and –denuded pulmonary artery rings from SHR presented with similar vasodilating responses to leptin (100 nmol/L) after endothelin-1 (10 nmol/L) precontraction (Figure 2D). Leptin-induced vasodilating response was more pronounced in aortic compared to pulmonary artery rings (Figure 2C and D).

### Vasoreactivity of arteries from spontaneously hypertensive and controls rats: response to leptin

**Figure 3:**
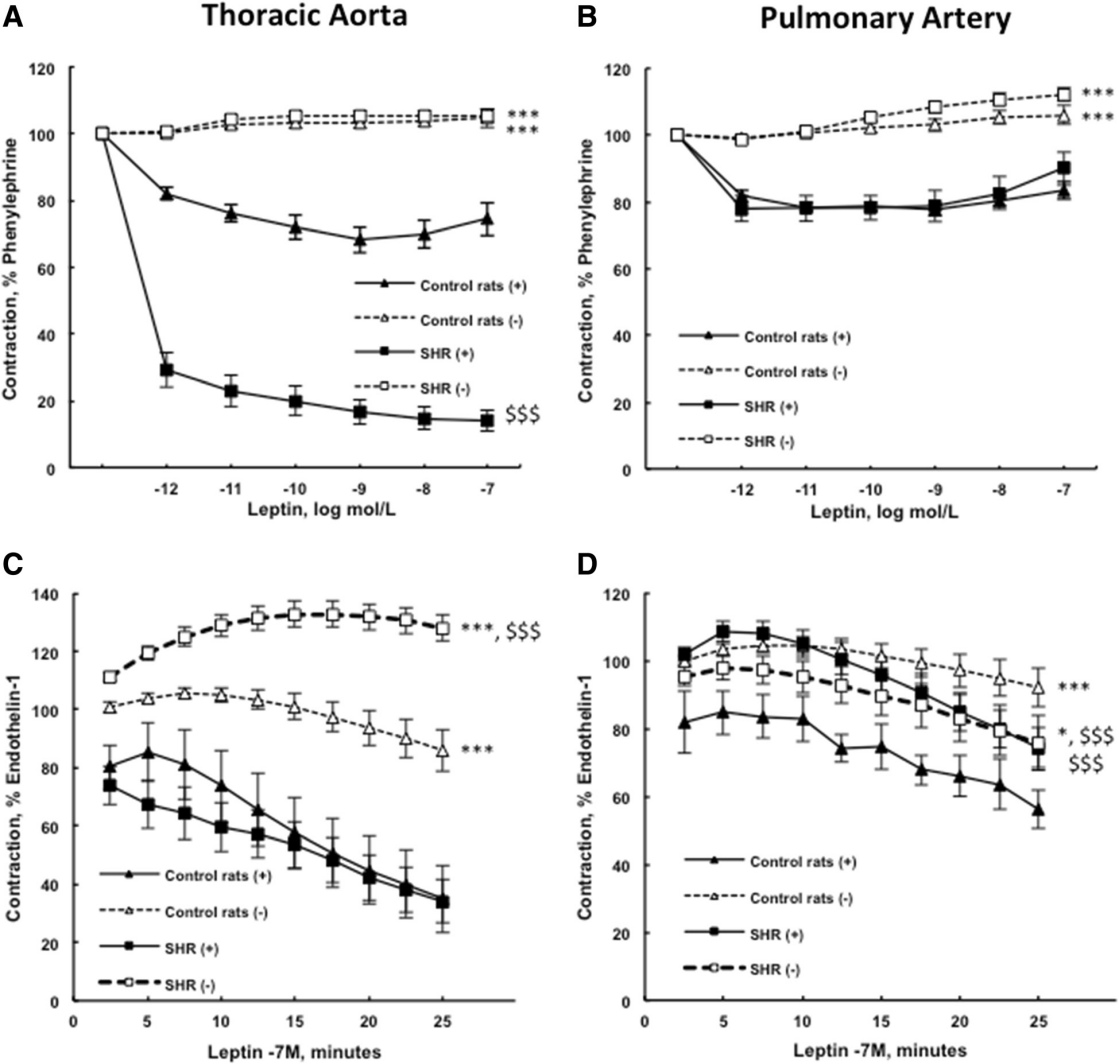
**Concentration-response curves to leptin after phenylephrine precontraction in spontaneously hypertensive compared to control Wistar rats.** The endothelium-denuded (−) and –intact (+) thoracic aortic **(A)** and pulmonary artery **(B)** rings were precontracted with 1 μmol/L phenylephrine before cumulative addition of leptin (0.001 to 100 nmol/L). Relaxation responses are expressed as the percentages of the maximal tension response obtained with 1 μmol/L phenylephrine. Response curves to leptin after endothelin-1 precontraction in spontaneously hypertensive rats compared to control Wistar rats. The endothelium-denuded (−) and –intact (+) thoracic aortic **(C)** and pulmonary artery **(D)** rings were precontracted with endothelin-1 (10 nmol/L) before addition of leptin (100 nmol/L). Relaxation responses are expressed as the percentages of the maximal tension response obtained with 10 nmol/L endothelin-1. Thoracic aortic and pulmonary artery segments are collected in control Wistar rats (n = 7-13) and in spontaneously hypertensive rats (n = 10-15). Results are expressed as means ± SEM. * 0.01 < p < 0.05, *** p < 0.001 compared to the corresponding endothelium-intact (+) artery; $$$ p < 0.001 compared to the corresponding artery from the control group.

As illustrated in Figure 3A, leptin (tested from 0.001 to 100 nmol/L) induced, in thoracic aortic rings from SHR, a stronger endothelium-dependent relaxation after phenylephrine (1 μmol/L) precontraction compared to control aortic response. This effect was totally abolished in endothelium-denuded rings. After endothelin-1 (10 nmol/L) precontraction, leptin (100 nmol/L) induced a similar endothelium-dependent relaxation in aortic rings from SHR and controls. However, after endothelin-1 (10 nmol/L), leptin had no effects on the vasoreactivity of endothelium-denuded aortic rings from controls, while it induced the contraction of denuded rings from SHR (Figure 3C).

After precontraction with phenylephrine (1 μmol/L), leptin (tested from 0.001 to 100 nmol/L) induced, in pulmonary artery rings from SHR and control rats, a similar endothelium-dependent relaxation, which rapidly reached a plateau. This effect was totally abolished in endothelium-denuded pulmonary artery rings (Figure 3B). After endothelin-1 (10 nmol/L) precontraction, leptin (100 nmol/L) induced an endothelium-dependent relaxation in pulmonary artery rings from control rats (p < 0.05), while this effect was decreased in pulmonary artery rings from SHR (Figure 3D).

### Pulmonary morphometry

**Figure 4:**
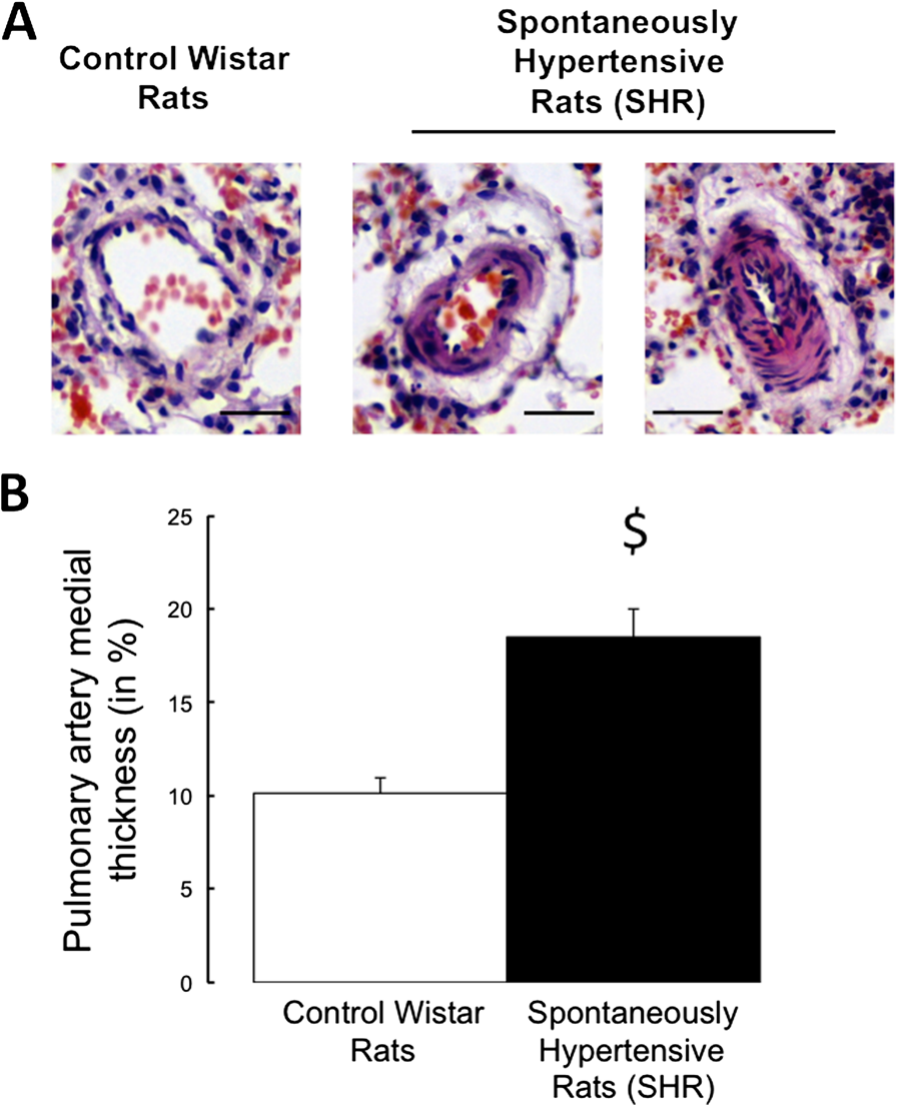
**Representative slides of pulmonary arterioles obtained from control Wistar and from spontaneously hypertensive rats.** Hematoxyllin-Eosin method staining (**A**; scale bars: 50 μm). Morphometry on pulmonary arterioles expressed as the medial thickness percentage versus the pulmonary artery external diameter in control Wistar rats (n = 6; white bars) and in spontaneously hypertensive rats (n = 6; black bars) pigs **(B)**. Values are expressed as mean ± SEM. $ 0.01 < p < 0.05 compared to the control pulmonary artery.

As illustrated in Figure 4, pulmonary artery medial thickness was increased in SHR compared to control rats.

### Pathobiological comparison of thoracic aorta and pulmonary arteries: Endothelin-1 and nitric oxide related molecules

**Figure 5:**
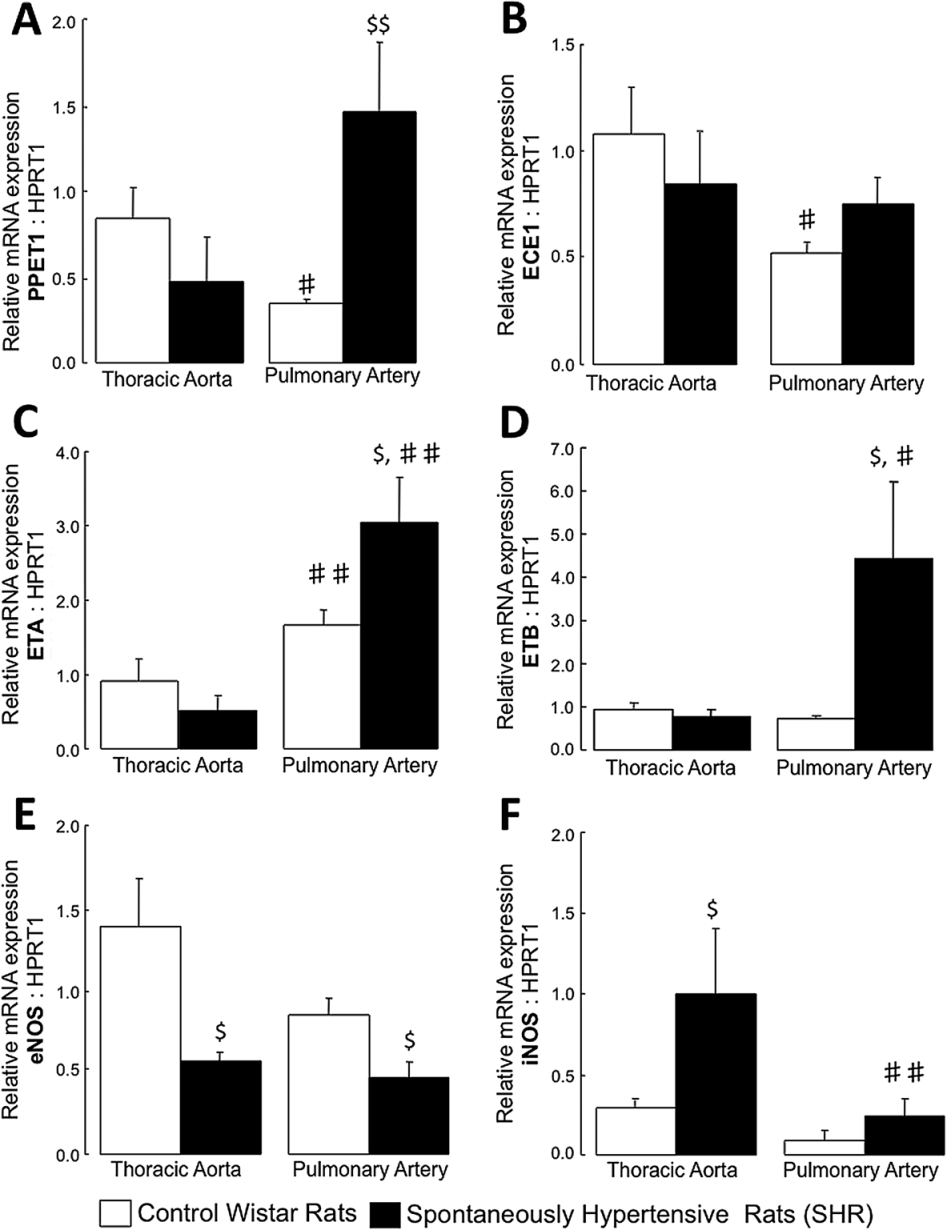
**Expression of genes implicated in the endothelin-1 and nitric oxide signaling pathways.** Relative gene expressions of the preproendothelin-1 (PPET-1, **A**), the endothelin converting enzyme-1 (ECE-1; **B**), the endothelin receptor type A (ET_A_; **C**), the endothelin receptor type B (ET_B_; **D**) and the endothelial (eNOS; **E**) and the inducible (iNOS; **F**) nitric oxide synthase in thoracic aorta and pulmonary artery from control Wistar rats and from spontaneously hypertensive rats (SHR). Results are expressed as means ± SEM. $ 0.01 < p < 0.05 compared to the corresponding artery from the control group; ♯ 0.01 < p < 0.05, ♯♯ 0.001 < p < 0.01 compared to the thoracic artery in the same strain of rats.

In control rats, gene expressions of the precursor of endothelin-1 (the preproendothelin-1; PPET-1) and of its converting enzyme (the endothelin converting enzyme-1; ECE-1) were lower in pulmonary arteries compared to aorta (Figure 5A-B), while endothelin-1 receptors type A (ET_A_) and type B (ETB) expressions were respectively higher and unchanged (Figure 5C-D).

In SHR, expressions of PPET-1, ET_A_ and ET_B_ receptors were increased in pulmonary arteries compared to aorta, while ECE-1 expression remained unchanged (Figure 5A-D). Gene expression of inducible nitric oxide (iNOS) was reduced in pulmonary arteries compared to aorta, while endothelial nitric oxide (eNOS) expression remained unchanged (Figure 5E-F).

In thoracic arteries, eNOS expression was decreased in SHR compared to controls, while expression of iNOS was increased (Figure 5E-F). In pulmonary arteries from SHR, expressions of PPET-1, ET_A_ and ET_B_ receptors were higher, while ECE-1 and iNOS expressions remained unchanged (Figure 5A-D and F). Pulmonary artery gene expression of eNOS was decreased in SHR compared to controls (Figure 5E).

### Thoracic aorta and pulmonary artery expressions of leptin and related molecules

**Figure 6:**
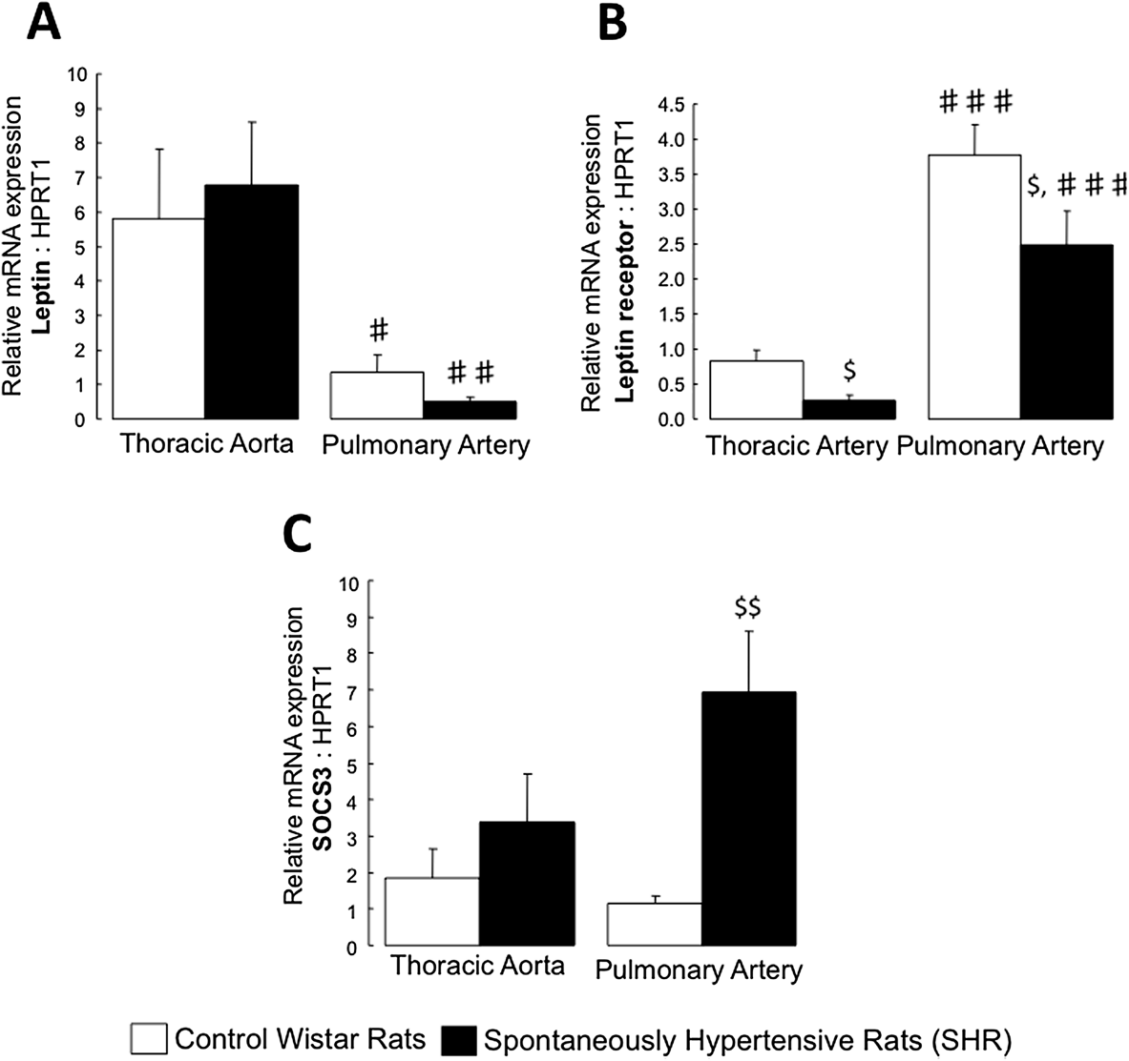
**Expression of genes implicated in the leptin signaling.** Relative gene expressions of the leptin **(A)**, the leptin receptor **(B)** and the suppressor of cytokine signaling 3 (SOCS3; **C**) in thoracic aorta and pulmonary artery from control Witar rats and spontaneously hypertensive rats (SHR). Results are expressed as means ± SEM. $ 0.01 < p < 0.05 compared to the corresponding artery from the control group; **♯** 0.01 < p < 0.05, **♯♯** 0.001 < p < 0.01, **♯♯♯** p < 0.001 compared to the thoracic artery in the same strain of rats.

In control rat and SHR, gene expression of leptin was lower in pulmonary artery than in thoracic aorta (Figure 6A), while leptin receptor gene expression was higher (Figure 6B).

In SHR, pulmonary artery and thoracic aorta expression of leptin receptor was decreased compared to controls (Figure 6B), while leptin expression did not change (Figure 6A). Gene expression of suppressor of cytokine signaling-3 (SOCS-3), which has been shown to inhibit the signal transduction processes of leptin and to be implicated in leptin resistance [18], was increased in pulmonary arteries from SHR compared to controls (Figure 6C).

## Discussion

The present study shows that vascular reactivity to endothelin-1 and leptin differs between pulmonary and systemic arteries and between pulmonary arteries from spontaneously hypertensive and normotensive rats. Indeed, the vasoconstriction induced by endothelin-1 is stronger in pulmonary compared to systemic arteries, and leptin induces more endothelium-dependent vasorelaxation in systemic compared to pulmonary arteries. In spontaneously hypertensive rats, the pulmonary artery vasoconstriction induced by phenylephrine and by endothelin-1 is enhanced due to a loss of the endothelial negative modulation of vasoconstriction. Moreover, leptin-induced pulmonary artery vasodilation after endothelin-1 precontraction is decreased in this hypertensive strain of rats, while leptin-induced thoracic aortic vasodilatation is preserved.

In the present study, pulmonary and systemic arteries were different in terms of vascular reactivity to different vasoactive agents (endothelin-1 and leptin). This is consistent with previous report showing that the mechanisms underlying the regulation of the vascular tone are different in pulmonary and systemic arteries, including calcium sensitization, channel activation/opening and superoxide effects [19]. An important role for the endothelin system has been well established in pulmonary arterial hypertension with demonstrated beneficial effects of endothelin receptor antagonists in these patients [20]. In contrast, the use of endothelin receptor antagonists has been disappointing in systemic cardiovascular diseases [21]. This is consistent with the present results and with previous results [22], showing enhanced pulmonary vasoreactivity to endothelin-1 in experimental heart failure. Here, high pulmonary vasoreactivity to endothelin-1 was associated with increased expression of ET_A_ receptor in pulmonary compared to systemic arteries, while ET_B_ receptor expression was similar. Indeed, it appears that the stimulation of the ET_A_ receptors on the smooth muscle cells causes vasoconstriction that is opposed by stimulation of the ET_B_ receptors present on the endothelial cells.

Leptin is a multifactorial adipose-derived adipokine that plays a critical role in bodyweight homeostasis and energy expenditure. Plasma leptin levels are markedly increased in obesity and associated metabolic syndrome. However, leptin levels have also been shown to be increased in patients with systemic hypertension [6,7] and pulmonary arterial hypertension [13], independently of their body mass index. The effects of leptin on systemic vasculature have been previously reported [23-26], but there is still no data regarding the pulmonary vascular effects of leptin. Under physiological conditions, leptin has no effect on systemic blood pressure, probably because its sympathetic nervous system stimulation is counteracted by depressor mechanisms, including endothelium–dependent vasorelaxation by endothelial nitric oxide and endothelium-derived hyperpolarizing factor. In contrast, in pathological chronic hyperleptinemia, the NO-mediated vasodilatory effects are impaired contributing to the development of hypertension, together with oxidative stress and overproduction of endothelin-1. Here, we found an endothelium-dependent vasodilating effect of leptin on isolated precontracted control systemic and pulmonary artery rings, with higher magnitude in systemic ones. This suggests that the systemic circulation is more prone to relax after leptin stimulation than the pulmonary circulation and that the endothelial integrity is critical for leptin vasodilation in both types of vessels. The decreased vasodilating response to leptin observed in the pulmonary arteries was associated with increased ET_A_ receptor expressions together with increased leptin receptor expression. Indeed, despite a higher gene expression of leptin receptor in pulmonary artery, aortic rings were more responsive to leptin than pulmonary artery rings.

Beside systemic hypertension, spontaneously hypertensive rats have been shown to develop pulmonary hypertension spontaneously [27] or after exposure to chronic hypoxia [28]. In the pulmonary artery from these hypertensive rats, we found increased expressions of the endothelin-1 precursor, ET_A_ and ET_B_ receptors and decreased expression of endothelial NO synthase. These alterations of the vasodilator/constrictor balance (in favor of increased vasoconstriction) present some similarities with those observed in patients with pulmonary arterial hypertension [21,29-31]. Moreover, these pathobiological anomalies were associated with a slight but significant pulmonary artery remodeling. We also found increased pulmonary artery constriction to phenylephrine and endothelin-1 in hypertensive rats, due to the loss of the endothelial buffering of vasoconstriction. These pathobiological and functional alterations strongly suggest altered endothelial function in the pulmonary circulation of these hypertensive rats, probably responsible for the development of pulmonary hypertension. This may be considered as a type of pre-capillary component of pulmonary hypertension that in conjunction with the passive component due to the backward transfer of the increased pulmonary venous pressure participates to the onset of pulmonary hypertension.

Endothelial dysfunction is an important factor in the pathogenesis of pulmonary arterial hypertension [29]. In our experiments, endothelial function may be easily estimated by the difference in contraction between endothelium-denuded and -intact rings. In the present study, the endothelial blunting effect to vasoconstrictors (phenylephrine and endothelin-1) was lost in the pulmonary arteries from hypertensive rats, suggesting pulmonary endothelial dysfunction. This has already been shown in systemic aortic vessels in SHR, with variable magnitude with aging [32]. Indeed, healthy aortic endothelium negatively regulates α1-agonists- and endothelin-1-induced contraction through a basal endothelial NO release [33-35], which is partly lost in spontaneously hypertensive rats [36-38]. The imbalance between NO and endothelin-1 signaling pathways may, at least, partially mediate pulmonary endothelial dysfunction in this hypertensive strain, together with previously described altered calcium handling [2]. Even if we found some similar pathobiological anomalies within the pulmonary circulation of SHR compared to those observed in PAH patients, there is no clear evidence that these two conditions share common early pathogenic mechanisms.

In the present study, leptin-induced pulmonary artery vasodilation after endothelin-1 precontraction was decreased in spontaneously hypertensive rats, suggesting that, in these rats, an impairment of leptin negative modulation against endothelin-1 vasoconstriction could participate to the increase in pulmonary vascular tone. In pathologic conditions of chronic hyperleptinemia, resistance to the vasodilatory effects of leptin has already been described in the systemic circulation [26,39] and incriminated in the development of systemic arterial hypertension owing to unopposed stimulation of the sympathetic nervous system by this hormone. This has been attributed to an abrogated effect of leptin on NO release [26,40]. Resistance to leptin may result from different mechanisms, including an imbalance between leptin and leptin-binding proteins in the blood, a receptor downregulation or polymorphism, and post-receptor defects [41]. Suppressor of cytokine signaling 3 (SOCS3) is a critical negative feedback regulator of leptin receptor signaling that has been shown to contribute to the development of leptin resistance through inhibition of signal transduction [42]. In the present study, leptin receptor expression was downregulated and SOCS3 expression was upregulated in the pulmonary arteries of spontaneously hypertensive compared to normotensive rats. These could, therefore, contribute to the *leptin resistance* observed in the pulmonary vasculature of the hypertensive rats.

## Conclusions

The present study shows that endothelin-1-induced vasoconstriction is more potent in the pulmonary artery than in the aorta and that this constriction is potentiated in spontaneous hypertensive rats, probably due to pulmonary endothelial dysfunction. The endothelium-dependent vasodilation induced by leptin is less effective in the pulmonary artery than in the aorta and this effect is abrogated after endothelin-1 precontraction in spontaneously hypertensive rats. The fact that this could be related to leptin resistance needs further investigation.

## Abbreviations

ECE-1: Endothelin-converting enzyme 1; ETA: Endothelin receptor type A; ETB: Endothelin receptor type B; HPRT1: Hypoxanthine-guanine PhosphoRibosylTransferase; NO: Nitric oxide; eNOS: endothelial nitric oxide synthase; iNOS: inducible nitric oxide synthase; PA+: Pulmonary artery with Endothelium; PA-: Pulmonary Artery without Endothelium; PPET-1: Preproendothelin-1; PVR: Pulmonary vacsular resistance; RTQ-PCR: Real-time quantitative polymerase chain reaction; SEM: Standard error of the mean; SHR: Spontaneously hypertensive rat; SOCS3: Suppressor of cytokine signaling 3; TA+: Thoracic aorta with endothelium; TA-: Thoracic aorta without endothelium.

## Competing interests

The authors declare that they have no competing interest.

## Authors’ contributions

Study conception and design: SG, CD, PJ, KM, LD - acquisition of data: SG, CD, PJ, MM - analysis and interpretation of data: SG, CD, PJ, MM, CM, KM, LD - drafting of manuscript: SG, RN, KM, LD - critical revision: SG, NL, SP, GB, RN, KM, LD. All authors read and approved the final manuscript.
